# The complete chloroplast genome of *Ulmus mianzhuensis* with insights into structural variations, adaptive evolution, and phylogenetic relationships of *Ulmus* (Ulmaceae)

**DOI:** 10.1186/s12864-023-09430-1

**Published:** 2023-06-29

**Authors:** Nan Lin, Rui Liu, Yakun Wang, Peng Guo, Yihan Wang, Yanpei Liu, Fude Shang

**Affiliations:** 1 College of Life Science, Henan Agricultural University, Zhengzhou, China; 2 Henan Engineering Research Center for Osmanthus Germplasm Innovation and Resource Utilization, Henan Agricultural University, Zhengzhou, China

**Keywords:** Chloroplast genome, *Ulmus*, Structural comparison, Phylogenomics, SSRs

## Abstract

**Background:**

*Ulmus mianzhuensis* is an endemic tree species in China with high ornamental and economic value. Currently, little is known regarding its genomic architecture, phylogenetic position, or adaptive evolution. Here, we sequenced the complete chloroplast genome (cp genome) of *U. mianzhuensis* and further compared the variations in gene organization and structure within *Ulmus* species to define their genomic evolution, then reconstructed the phylogenomic relationship of 31 related *Ulmus* species to explore the systematic position of *U. mianzhuensis* and the utility of cp genome for resolving phylogenetics among *Ulmus* species.

**Results:**

Our results revealed that all the *Ulmus* species exhibited a typical quadripartite structure, with a large single copy (LSC) region of 87,170 − 88,408 bp, a small single copy (SSC) region of 18,650 − 19,038 bp and an inverted repeat (IR) region of 26,288 − 26,546 bp. Within *Ulmus* species, gene structure and content of cp genomes were highly conserved, although slight variations were found in the boundary of SC/IR regions. Moreover, genome-wide sliding window analysis uncovered the variability of *ndhC-trnV-UAC*, *ndhF-rpl32*, and *psbI-trnS-GCU* were higher among 31 *Ulmus* that may be useful for the population genetics and potential DNA barcodes. Two genes (*rps15* and *atpF*) were further detected under a positive selection of *Ulmus* species. Comparative phylogenetic analysis based on the cp genome and protein-coding genes revealed consistent topology that *U. mianzhuensis* is a sister group to *U. parvifolia* (sect. *Microptelea*) with a relatively low-level nucleotide variation of the cp genome. Additionally, our analyses also found that the traditional taxonomic system of five sections in *Ulmus* is not supported by the current phylogenomic topology with a nested evolutionary relationship between sections.

**Conclusions:**

Features of the cp genome length, GC content, organization, and gene order were highly conserved within *Ulmus*. Furthermore, molecular evidence from the low variation of the cp genome suggested that *U. mianzhuensis* should be merged into *U. parvifolia* and regarded as a subspecies of *U. parvifolia*. Overall, we demonstrated that the cp genome provides valuable information for understanding the genetic variation and phylogenetic relationship in *Ulmus*.

**Supplementary Information:**

The online version contains supplementary material available at 10.1186/s12864-023-09430-1.

## Background

Elm (*Ulmus* L.) is a representative genus of Ulmaceae including about 40 species, which is mainly distributed in the temperate regions of the Northern Hemisphere [1–3]. China has been regarded as one of the original and divergent centers of *Ulmus* owning more than 25 species. In addition, there are at least 10 species of *Ulmus* restricted to small regions and almost half of them are endemic to China [1]. For instance, *U. elongata* is regarded as the ancient Tertiary species and extremely small populations, and *U. macrocarpa*, *U. lamellosa* as well as *U. lanceifolia* are listed as nationally protected and endangered species in China [4]. Elm trees have the characteristics of high ornamental value, fast growth, and wide adaptability due to their strong resistance to abiotic stress, such as drought and cold [5]. In addition, its bark, leaf and fruit have been widely used as medicine to calm nerves and diuretics, with immeasurable development prospects of raw material for the chemical industry [6]. According to flowering phase, inflorescence, and samara type, a complete infrageneric classification of the world *Ulmus* species was built recently, in which *Ulmus* was divided into five sections including sect. *Blepharocarpa* Dumert, sect. *Chaetoptelea* Schneid, sect. *Microptelea* Benth, sect. *Trichoptelea* Schneid, and sect. *Ulmus* [1–3, 7, 8]. Specifically, sect. *Microptelea* and sect. *Trichoptelea* are distinguished from other species by flowering phase in the fall to winter rather than spring. The sect. *Blepharocarpa* is named by its samara pubescent in the margin, while peduncles slender of sect. *Chaetoptelea* are elongated and particularly pendulous. The sect. *Ulmus* with more than 20 species is extremely diverged, which is comprised of four series (Ser. Lanceifoliae, Ser. Nitentes, Ser. Glabrae, and Ser. Villosa) [8]. However, a wide range, similar ecological affinities and morphological characters, and recent diversification caused it is difficult to discriminate *Ulmus* species based on morphological variations independently. Thus, an intensification of taxonomic and systematic research is still needed for *Ulmus* species. Previous molecular evidence based on several fragments (e.g., *ITS*, *atpB-rbcL*) or limited cp genomes has largely enhanced our understanding of phylogeny in *Ulmus* [8–11]. Wiegrefe et al. (1994) established the phylogenetic backbone of the *Ulmus* based on restriction sites, dividing five sections into two subgenera: subg. *Oreoptelea* (sects. *Blepharocarpa*, *Chaetoptelea*, and *Trichoptelea*) and subg. *Ulmus* (sects. *Microptelea* and *Ulmus*) and assigning *U. lanceifolia* to sect. *Lanceifoliae*. Latterly, based on DNA fragments and cp genomes, resolution of phylogeny in *Ulmus* was largely improved, but the relationships among sections and within the sect. *Ulmus* were still unresolved [9, 10, 12]. Until now, species delimitation and phylogenetic relationships within this genus have been improperly addressed due to the rapid radiations and hybridization [13]. Therefore, a further study based on genomic information is crucial to explore the phylogenetic evolution of *Ulmus* and accelerate the effective conservation and utilization of *Ulmus* germplasm.

*Ulmus mianzhuensis* is an endemic species from *Ulmus* which is distributed in Sichuan, China [14]. Based on the morphological trait, *U. mianzhuensis* is assigned to the sect. *Microptelea* as flowering phase of this section usually appears in autumn. Currently, sect. *Microptelea* is composed of two species, *U. crassifolia* and *U. parvifolia.* Geographically, *U. mianzhuensis* can be easily discriminated from the other two, because *U. parvifolia* is mainly distributed in East Asia, and *U. crassifolia* is restricted to North America [2]. The variability of bark color, petioles, and pedicel length have been recognized as the main keys of taxonomy among the different species. Specifically, the bark is grey and light brown in *U. parvifolia* and *U. crassifolia* respectively, but it is dark gray in *U. mianzhuensis.* Petioles length is very short (less than 2 mm) in *U. crassifolia*, but it is 2–6 mm and 3–5 mm in *U. parvifolia* and *U. mianzhuensis* respectively. Besides, the pedicel length of *U. parvifolia* is over 8 mm, which is short in other species. A previous study suggested that *U. mianzhuensis* is highly similar to *U. parvifolia* in identical flowering phase, pale pubescent at the stigma of samara and seed locating the middle to upper of samara. Whereas, molecular systematics is still sparse to determine the taxonomic status of *U. mianzhuensis* and the relationship between *U. mianzhuensis* and relative species remains uncertain.

Due to uniparental inheritance, and large copy numbers in plant cells without recombination, chloroplast genome has been widely applied in phylogenomic studies in the recent years, resolving deep relationships of particularly recalcitrant lineages undergoing recent radiations [15–17]. Previous studies demonstrated that the structural variation and abundant phylogenetic information of cp genomes are useful to solve the evolutionary relationship of complicated species [18, 19]. Although the cp genome size and gene structure are usually conserved, it can also provide insights into the molecular evolutionary patterns to help with species discrimination. For example, comparative studies showed that structural variation also occurred at the genus level and presented specific phylogenetic signals, such as *Amphilophium*, *Corylus* and *Epimedium* [20–22]. Except for structural variations among species, the adequate evolutionary information in cp genome sequences can significantly improve the resolution of deep phylogenetic relationships, such as the backbone tree of major angiosperms, and the identification of closely related species in *Rhododendron* and *Pimelea* [23, 24]. Therefore, combining structural variation and phylogenetic information in cp genome provides an important approach for the interspecific phylogenetic relationship and identification. In the present study, we sequenced the complete chloroplast genome of *U. mianzhuensis* and the objectives of this study were as follows: (1) to investigate the interspecific structural variation in the cp genome of *U. mianzhuensis* and other related species from the genus *Ulmus*; (2) to screen highly variable hotspot regions and simple sequence repeats candidate sequences for species identification and genetics resources; (3) to resolve the systematic position of *U. mianzhuensis* and reconstruct phylogenetic relationships between *U. mianzhuensis* and relative species among *Ulmus* based on cp genomes. These results will enhance our understanding of the evolution of the genus *Ulmus* and its close relatives.

## Results

### Organization and features of chloroplast genome

**Fig. 1:**
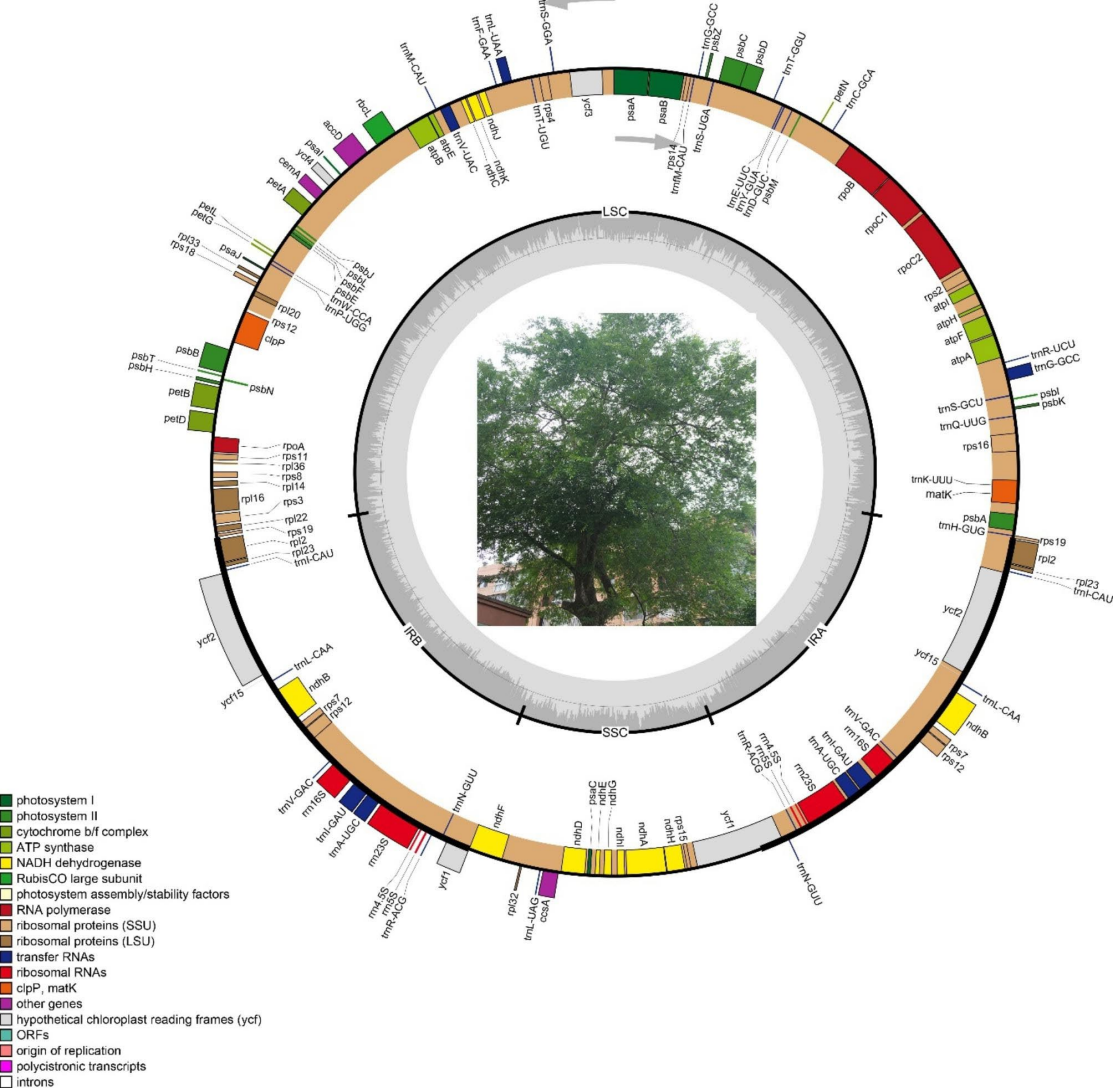
Physical maps of *Ulmus mianzhuensis* chloroplast genome. The direction of transcription is indicated by arrows. The gene function is color-coded as shown in the legend. The darker gray in the inner circle shows the GC content, while the lighter gray shows the AT content. LSC, large single-copy region; SSC, small single-copy region; IRa, IRb, inverted repeat A and B, respectively

Our results found that *U. mianzhuensis* and all the relative species of *Ulmus* possessed a typical quadripartite structure consisting of a pair of identical IRs separated by LSC and SSC regions (Fig. 1). The genome size of the 31 *Ulmus* species ranged from 158,742 bp (*U. lanceifolia*) to 159,795 bp (*U. microcarpus*). The largest LSC, SSC, IR were found in *U. microcarpus* (88,408 bp), *U. macrocarpa* (19,038 bp) and *U. mianzhuensis* (26,546 bp), while the smallest LSC, SSC and IR were found in *U. lanceifolia* (87,170 bp), *U. laevis* (18,650 bp) and *U. pumila* (26,288 bp), respectively (Table S1). Besides, all the cp genomes from *Ulmus* shared the same GC content of 35.5%. A total of 113 unique genes, comprising 79 protein-coding genes, 30 tRNA genes, and 4 rRNA were enrolled in *Ulmus* species (Fig. 1). Among the 113 distinct genes, 15 genes (*trnK-UUU*, *rps16*, *atpF*, *rpoC1*, *trnL-UAA*, *ndhB*, *trnA-UGC*, *ndhA*, *trnG-GCC*, *trnV-UAC*, *trnI-GAU*, *rpl2*, *rpl16*, *petB*, *petD*) contained one intron and three genes contained two introns (*ycf3*, *clpP*, *rps12*) in the *Ulmus* cp genomes.

### Comparison of chloroplast genome among *Ulmus* species

**Fig. 2:**
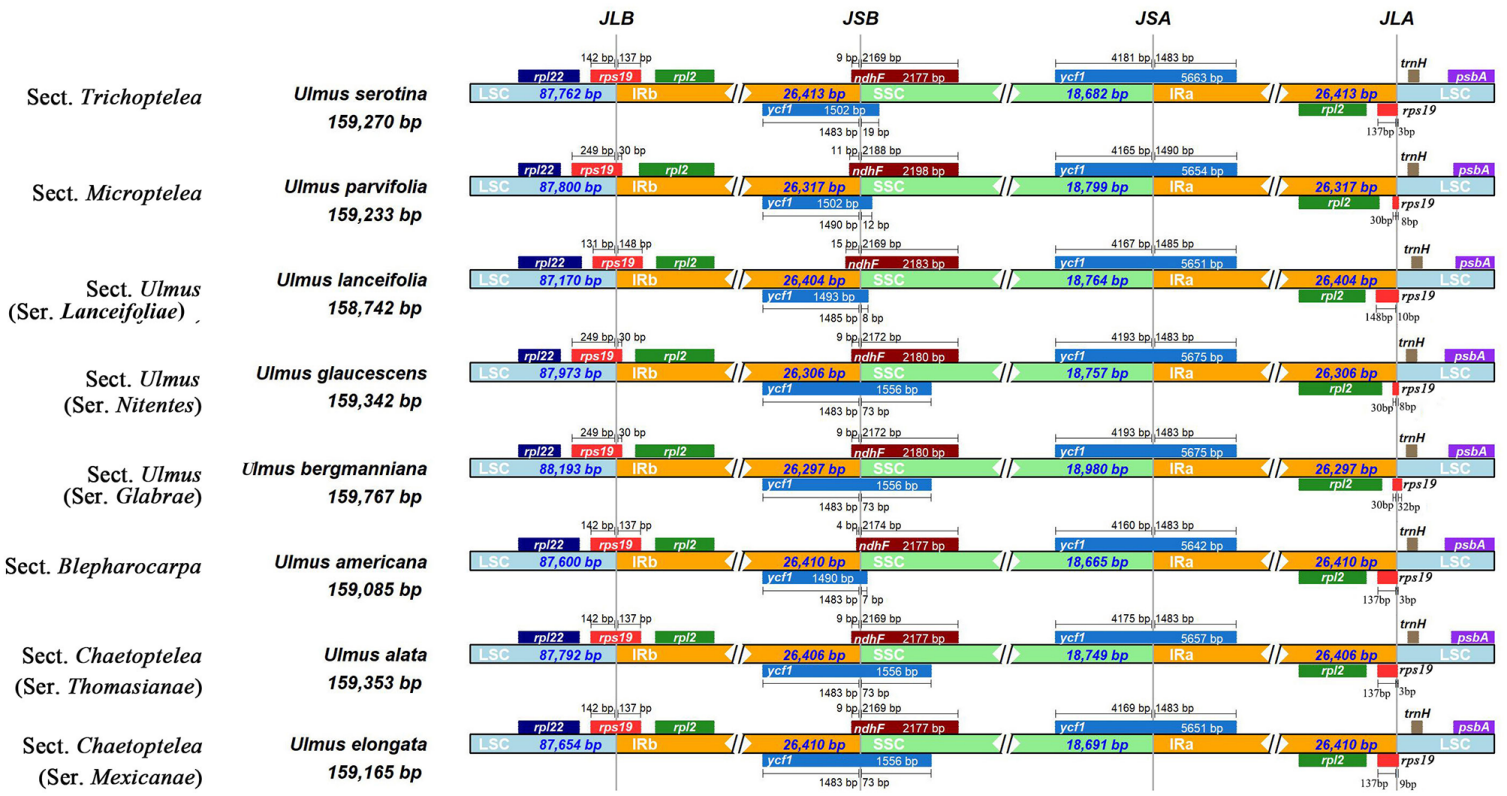
Chloroplast region (IR/SSC/LSC) junctions of eight representative *Ulmus* chloroplast genomes from five sections and different series. JSA: junction between SSC and IRa; JSB: junction between SSC and IRb; JLA: junction between LSC and IRa; JLB: junction between LSC and IRb

**Fig. 3:**
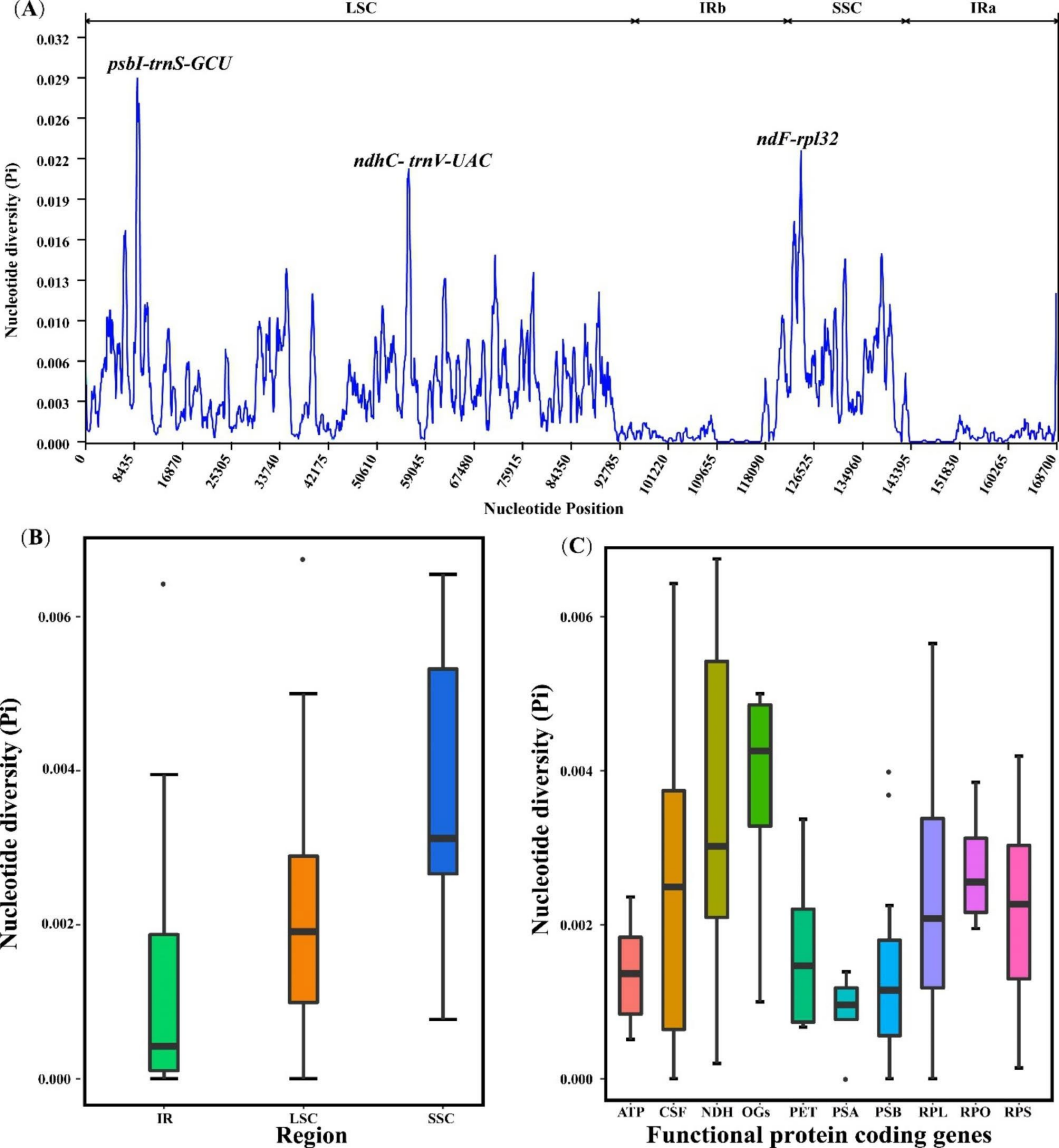
**(A)** Sliding-window analysis of the chloroplast genomes for *Ulmus* species. The window length is set as 600 bp with the step size as 200 bp. The X-axis corresponds to the midpoint position of a window and Y-axis shows the nucleotide diversity (Pi) of each window; **(B)** The nucleotide diversity (Pi) in chloroplast regions (IR/SSC/LSC); **(C)** The nucleotide diversity (Pi) of different functional groups and the detailed information for functional groups could be found in Table S3

Based on our results, gene content and order were relatively conserved among the *Ulmus* cp genomes, and no rearrangement occurred in gene organization (Fig. 2). The divergence of cp genomes plotted using the mVISTA program presented lower sequence divergence in IR regions than in SC regions (Fig. S1). When compared to the diversity of nucleotide divergence between *U. mianzhuensis* and *U. parvifolia*, our result showed a relatively low-level nucleotide variation of the cp genome (0.0003). The nucleotide divergence for all *Ulmus* cp genomes ranged from 0 to 0.02876, and the hotspots variation region with the divergence over 0.025 was found in *ndhC-trnV-UAC*, *ndhF-rpl32*, *psbI-trnS-GCU* (Fig. 3A). Meanwhile, the average nucleotide diversity in the SC region is higher than that in the IR region, which is same as shown in mVISTA (Fig. 3B). We further found the OG groups had the highest median value of nucleotide variation from the functional groups for all protein-coding genes, while genes associated with ATP synthase (ATP), photosystems I (PI) and photosystems II (PII) had a lower nucleotide diversity (Fig. 3C). Comparison of adjacent genes among the *Ulmus* species uncovered genes *ycf1* was located in the junction of IRb/SSC and *rps19* was located in IRa/LSC boundary. Nevertheless, the genes in the IR border position presented slight variations among these *Ulmus* species (Fig. 2). The *ycf1* gene occurring on the SSC/IRb boundary weakly varied with 1483–1490 bp located in the IRb region across sections of *Ulmus.* Similarly, the *rps19* occurring on the IRa/LSC boundary had 30–148 bp located in the IRa region.

### Simple sequence repeats and long repeats sequences analysis

**Fig. 4:**
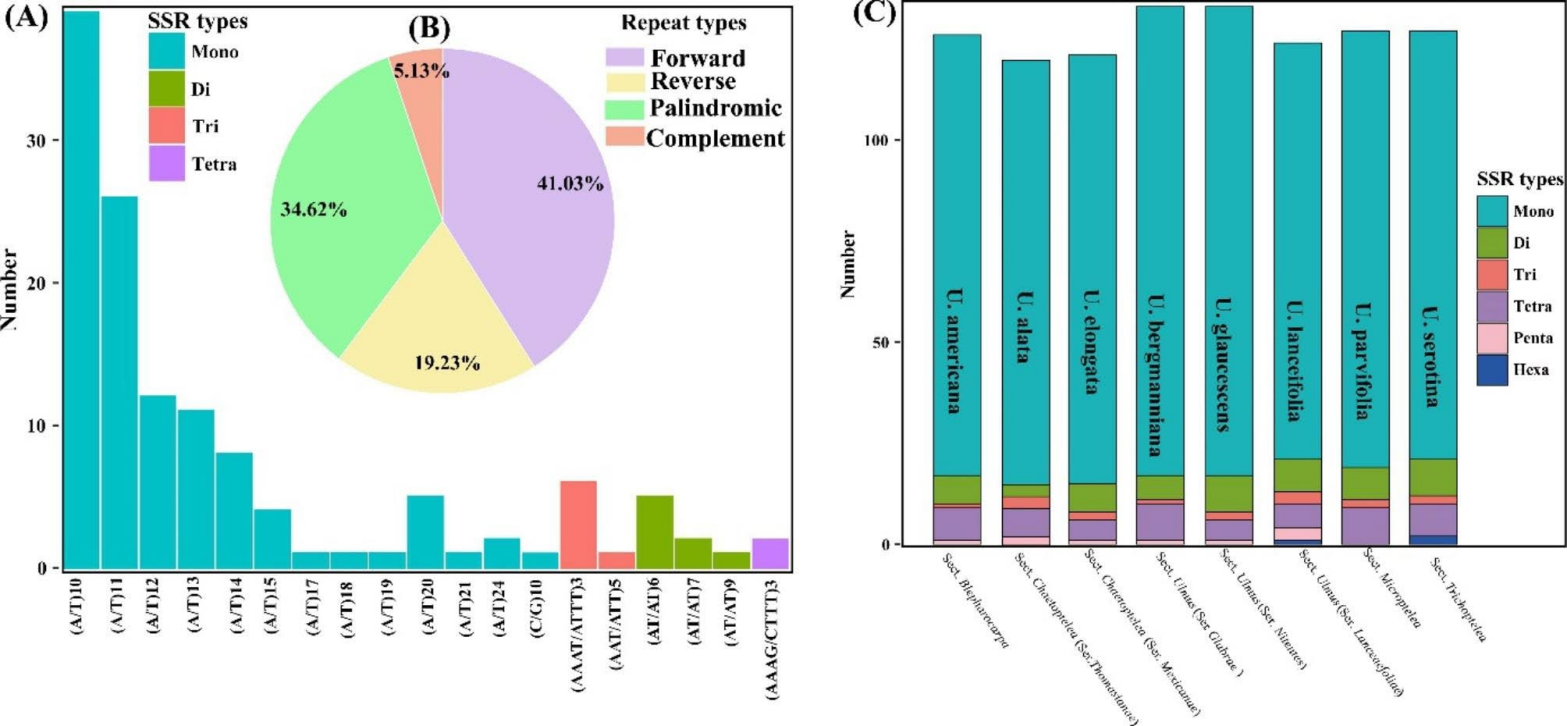
**(A)** The type and distribution of SSRs in the *U. mianzhuensis* chloroplast genome. **(B)** The proportion of four repeats types in *U. mianzhuensis* chloroplast genome; **(C)** Number of identified SSR repeat types in the eight representative *Ulmus* chloroplast genomes from five sections and different series

A total of 129 simple sequence repeats (SSRs) were identified in *U. mianzhuensis* mainly composed of four types of SSRs (Fig. 4). Among these SSRs, the majority of SSRs were mononucleotides (86.82%), eight were dinucleotide repeats (6.20%), eight were tetranucleotide (6.20%), whereas only one was trinucleotide repeat (0.08%). We also identified different types of repeats sequences within *U. mianzhuensis*, about 41.02% were forward repeats, 34.62% were palindromic repeats, 19.23% were reverse repeats, and only 5.13% were complement repeats screened in *U. mianzhuensis* (Fig. 4B). Furthermore, the SSRs distribution among species of representative sections showed the same trend in types and number of SSRs (Fig. 4C). A total of 120 ~ 133 SSRs were identified among these species, and the highest and lowest number of SSRs were found in *U. bergmanniana*/*U. glaucescens* and *U. alata* respectively. Among these SSRs, over 80% were mononucleotides, and hexanucleotides were only found in *U. serotina* and *U. lanceifolia*.

### Phylogenomic analysis and PCGs substitution rates within *Ulmus*

**Fig. 5:**
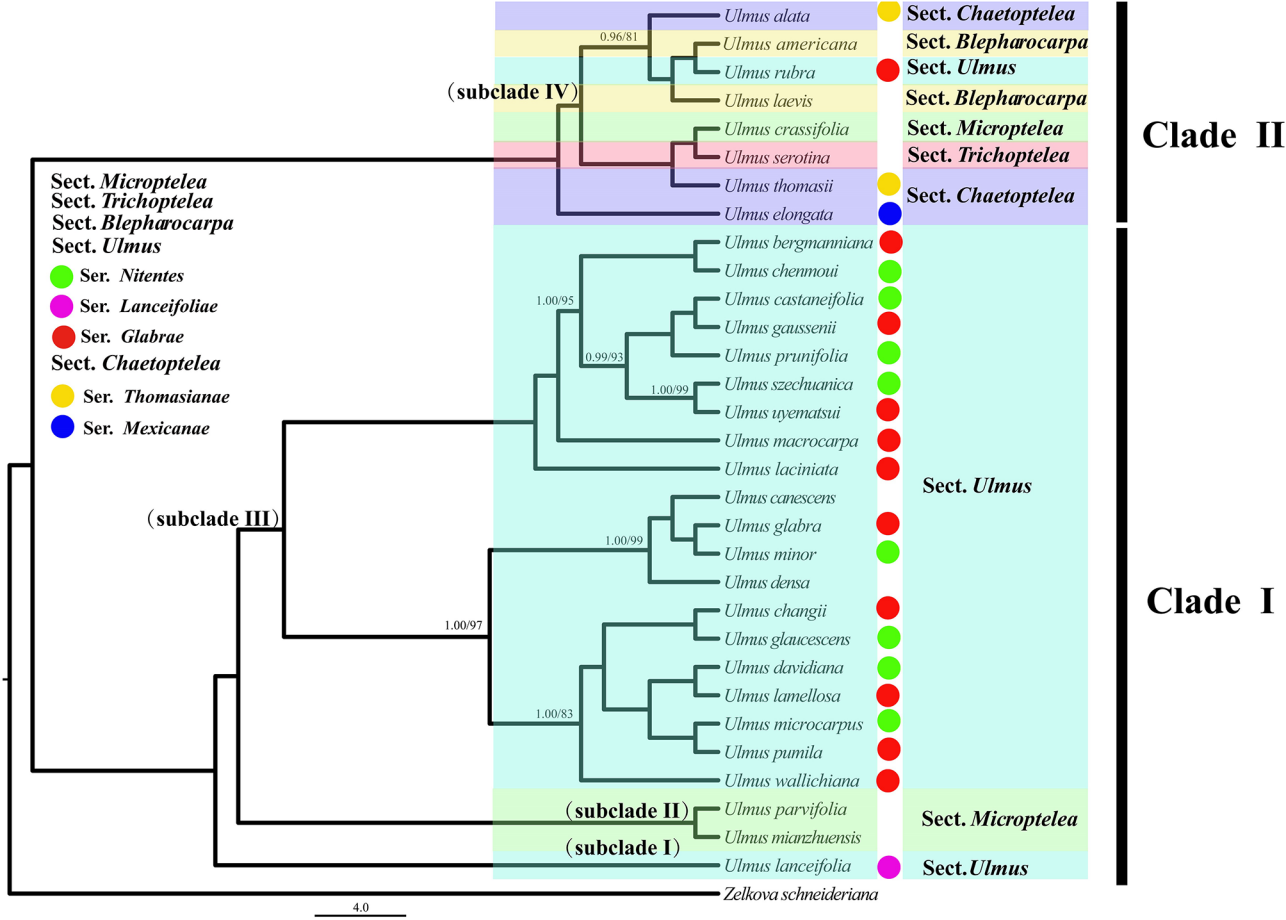
Phylogram of Maximum likelihood (ML) tree of *Ulmus* based on chloroplast genomes. Only the posterior probabilities (PP) and likelihood bootstrap (BS) values were separated by a slash and only the support values less than 100/1.00 was shown at nodes

**Fig. 6:**
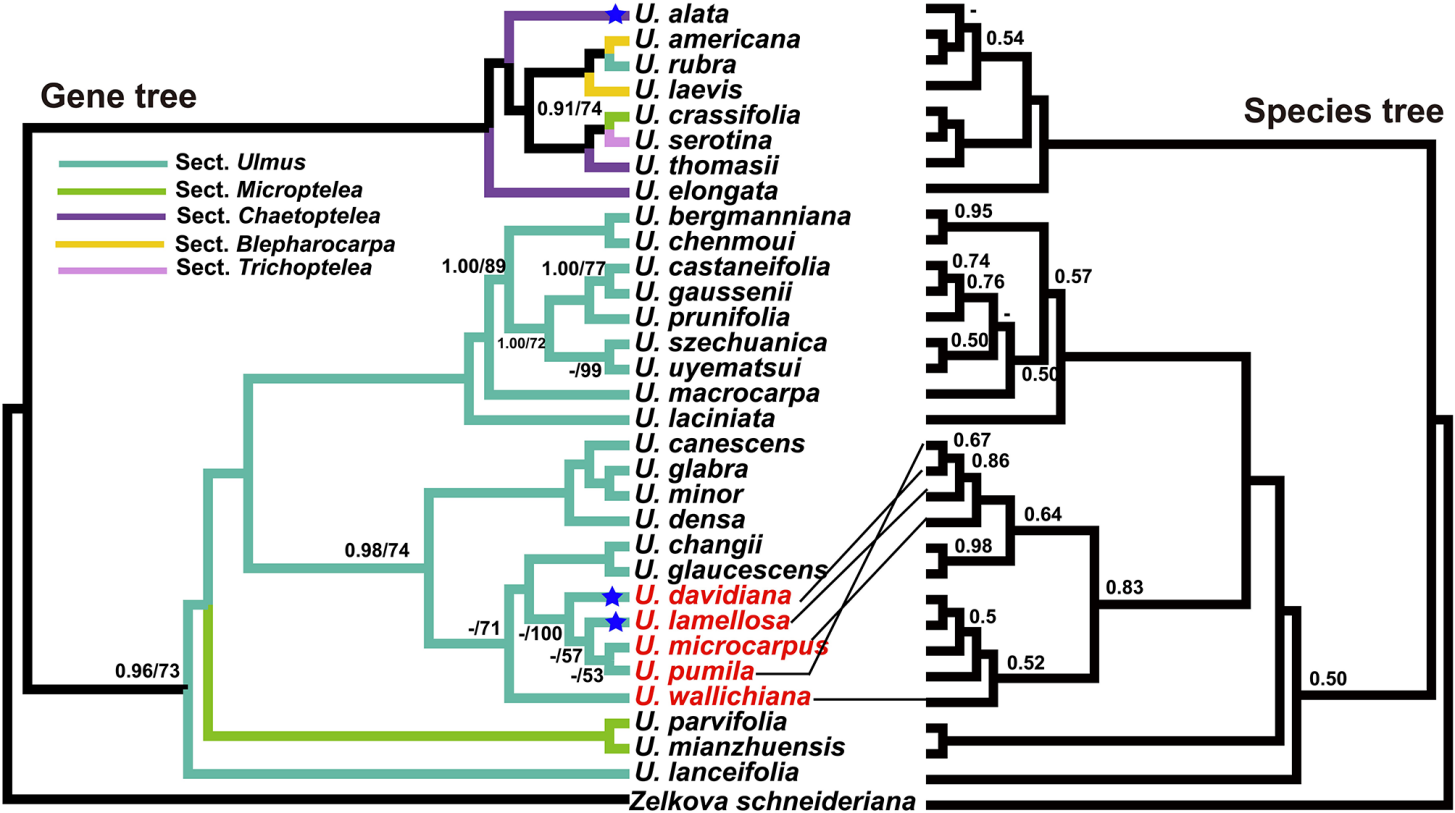
Comparison of the topology of *Ulmus* based on plastid protein-encoding genes. The left is a phylogenetic tree based on concatenated genes based on ML and BI methods. Only the maximum likelihood bootstrap (BS) values and the posterior probabilities (PP) less than 100/1.00 was shown at nodes, ‘-’ indicates the support less than 50/0.50, the tip labels with a blue star showing the topology conflicts between chloroplast genome and protein-encoding genes; (b) phylogeny from the coalescent method in ASTRAL-III, and only local posterior probabilities (LPP) below 1.00 was shown in the tree. Conflicted taxa are highlighted in red font and connected by black lines between two phylogenetic trees

**Fig. 7:**
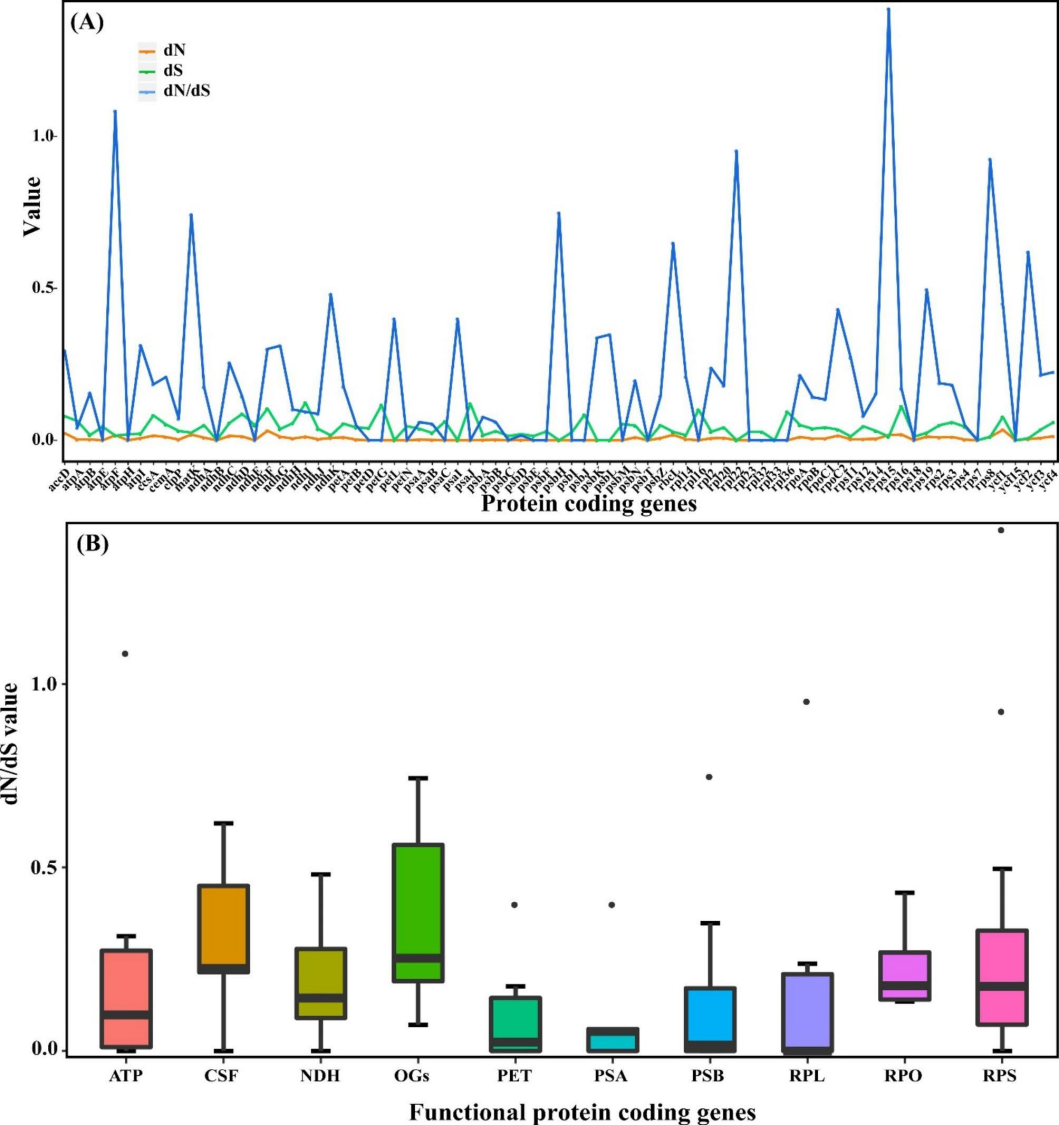
**(A)** The nonsynonymous (dN), synonymous (dS) substitution rates and dN/dS value of all plastid protein-coding genes (PCGs) based on PAML; **(B)** dN/dS value of different functional protein-coding genes (PCGs) groups

Base on the 32-taxon data matrix, we obtained a well-resolved and highly consistent phylogeny including the two main clades of *Ulmus* via ML and BI methods (BS = 100, PP = 1; Fig. 5). From our phylogeny, five *Ulmus* sections could not be formed to the monophyletic groups, and similar results exhibited in the series level. For the clade I, there were three subclades including two species of sect. *Microptelea* and almost species of sect. *Ulmus* with *U. lanceifolia* in the basal group (BS = 100, PP = 1). Notably, our target specie *U. mianzhuensis* was clustered into subclade II together with *U. parvifolia* in sect. *Microptelea* (BS = 100, PP = 1). Within the subclade III of sect. *Ulmus*, the species were separated into two clusters, which were distributed irregularly at the series level. For the clade II, *U. elongata* from sect. *Chaetoptelea* was evolved independently from other two species (*U. thomasii* and *U. alata*; BS = 100, PP = 1), of which *U. thomasii* was clustered to *U. serotina* and *U. crassifolia*. The sect. *Blepharocarpa* did not appear monophyletic group with *U. rubra* embedded in it. For 79 concatenated protein-coding genes, the partitioned scheme has obtained highly congruent phylogeny with cp genome of strongly supported bootstrap values (Figs. 5 and 6). Whereas, the systematic position for *U. alata*, *U. davidiana* and *U. lamellosa* is incongruent with the cp genome phylogeny (Fig. 6). When compared the concatenated tree to species tree from ASTRAL-III, we found they had highly consistent phylogenetic backbone of *Ulmus*, with *U. lanceifolia* resolved as an isolated clade (Fig. 6). There were still a few alternative topologies among species including *U. davidiana*, *U. microcarpus*, *U. pumila*, *U. lamellosa* and *U. wallichiana* with low support. The analyses of the selection pressure on the 79 protein-coding genes within *Ulmus* plastomes by CODEML indicated that two genes *atpF* and *rps15* were under positive selection (adaptive selection) (Fig. 7A). Except these genes, the other genes were probably under neutrality or purifying selection. The unknown (CSF) genes, DNA-dependent RNA polymerase genes (RPO), other genes (OGs) and small subunit of ribosome genes (RPS) had higher median values of dN/dS (Fig. 7B).

## Discussion

### The cp genome variations of *Ulmus* and its utility in DNA barcoding

In the current study, the cp genomes of gene content and order are highly conserved among the *Ulmus* species and all the *Ulmus* genomes shared the typical quadripartite architecture and the same GC content (35.5%). The junction genes located in SC/IRs boundaries were stable for *Ulmus* species with *rps*19/*ycf1* genes lying in LSC/IR and SSC/IR boundaries respectively that are identical with most angiosperm plants [25]. Previous studies found that IR expansion/contraction could alter the structural conservatism of the cp genomes, which partly explain the absence of rearrangement events and variations of the *Ulmus* [26, 27]. Significant differences in IR expansion/contraction have been reported in other genera (e.g., *Anemopaegma*, *Pedicularis*) that could be useful signals for species identification at an interspecific level [22, 28]. Moreover, this stability of *Ulmus* cp genomes might be due to its recent diversification, or related to the conservative ecological niches within the genus [1]. Variations in length occurring on the junctions of IR/SC regions between different *Ulmus* sections were still detected, just as we found the variation of nearly 1 kb between the smallest (sect. *Lanceifoliae*, *U. lanceifolia*) and largest (sect. *Ulmus*, *U. microcarpus*) genomes, respectively (Fig. 2). The cp genomes size is usually closely associated with the contraction/expansion of the IR region or gene losses as shown in previous studies [29]. For instance, extreme IR expansion has caused a higher genome size in *Pelargonium* [30] and lots of gene losses had shrunk chloroplast genome size in some parasitic plants and conifers [31–33]. Our result confirmed that IR contraction of SSC regions was responsible for the relatively smaller size of *U. lanceifolia* (sect. *Lanceifoliae*) and IR expansion of SSC regions was partly result in the larger size in *U. bergmanniana* (sect. *Ulmus*).

Complete cp genome sequences have been regarded as super barcodes and are increasingly used to solve taxonomic problems among closely related groups. Both structural variation and valuable evolutionary information from cp genome are sufficient to discriminate genetically close species [34–36]. Although the quadripartite architecture in our current study was highly conserved within *Ulmus*, abundant information in cp genome could provide sufficient and full resolution for species discrimination. Regardless of the fact that *trnH-psbA*, *rbcL* and *matK* have been recommended as the core barcodes in land plants, such as Calycanthaceae [37], Saxifragales [38] and ferns [39, 40]. Our analyses found three noncoding regions (*psbI-trnS-GCU*, *ndhC-trnV-UAC*, *ndhF-rpl23*) with high levels of genetic divergence between sections and species of *Ulmus* were effective to species distinguishing than the universal barcodes (Fig. 3). Thus, compared to the universal DNA barcoding, these variations hotspots exhibited higher solution for taxonomy in the *Ulmus* with priority to be used as future barcodes for species identification. Furthermore, cp genome data revealed great potential to resolve the phylogenetic relationships in the genus *Ulmus* with full resolution.

### Signature of positive selection on plastid genes

The evolutionary rate estimation based on dN/dS is widely used in phylogenetic and evolutionary studies. In most protein-coding genes, nucleotide substitutions of synonymous occurred more frequently than non-synonymous, with most of them were under neutral and purifying selection [41]. Overall, *Ulmus* species were subjected to a purifying selection, which helps to eliminate the disadvantageous mutations in populations evolution as the almost other genes (Fig. 7). However, our analyses also found that two genes were significantly under positive selection (dN/dS > 1) among the protein-coding genes within *Ulmus* (i.e., *atpF*, *rps15*), The *atpF* gene is one of photosystem subunit genes and participates in the encoding of the H^+^-ATP subunits, with essential roles in photosynthetic processes [42]. This gene has experienced positive selection in other land plants including Cucurbitaceae [43], Liliaceae [44] and Zingiberaceae [45], which functioned as the main source of energy for living cells and multicellular organisms. The *rps15* is a small ribosomal protein which involved in the regulation of chloroplast translation. Empirical test found that knockout of the *rps15* gene in tobacco have caused a definite reduction in small 30 S ribosomal subunits [46]. The positive pressure of *rps15* is also identified in Araliaceae [47], Fabaceae [48] and Rhizophoraceae [49], suggesting the important role to adapt to their living environment. The *Ulmus* species are widely distributed in East Asia and North America, and have been undergoing adaptive evolution in response to stressful environments in heterogeneous habitats. Aa a result, these positive selection genes will enrich the *Ulmus* variety and adaptability during long term evolution.

### The systematic position of *U. mianzhuensis* and reference to the phylogenomic relationship within *Ulmus*

Based on our phylogenomic results from cp genomes and protein-coding datasets, *U. mianzhuensis* was resolved as a sister group to *U. parvifolia* (Fig. 5; BS = 100, PP = 1), which demonstrated its systematic position in sect. *Microptelea*. Notably, nucleotide diversity between *U. mianzhuensis* and *U. parvifolia* showed low level variation of the cp. genome, suggesting that *U. mianzhuensis* might be merged into *U. parvifolia* and regarded as a subspecies of *U. parvifolia*. According to recent taxonomic systems [2, 8], sect. *Microptelea* is comprised of two species (*U. crassifolia* and *U. parvifolia*) and of which shared fascicle-cyme, equal pedicel and flowers appearing in fall. However, our phylogenomic analyses found that these species were not a monophyletic group, with *U. crassifolia* clustered to *U. serotina* (sect. *Trichoptelea*) (Fig. 5; BS = 100, PP = 1). Both *U. crassifolia* and *U. serotina* are mainly distributed in Southeastern North America, although they are differentiated in length and type of inflorescences. Therefore, our result suggested *U. crassifolia* should be separated from the sect. *Microptelea*, and merged to sect. *Trichoptelea*, which is consistent with previous molecular evidence [10]. Besides, the conflict between morphological taxonomy and phylogenetic topology was also found in sect. *Ulmus*, which was the most diverse group with the largest number of species. The particular species, *U. lanceifolia* has been basal species from the sect. *Ulmus*, which was only an evergreen tree and limited to Southeast Asia and South regions of China (Fig. 5; BS = 100, PP = 1). The position of *U. lanceifolia* has been slightly resolved in previous results, as Wiegrefe et al. (1994) and Fu (1980) has assigned it to sect. *Lanceifoliae* and sect. *Ulmus* successively. Our results revealed that *U. lanceifolia* should be independent from sect. *Ulmus* and supported the resumption of sect. *Lanceifoliae* rather than one of series of sect. *Ulmus*. We also found that *U. rubra* from sect. *Ulmus* was close to two species (*U. americana* and *U. laevis*) from sect. *Blepharocarpa* (Fig. 5; BS = 100, PP = 1). Morphologically, *U. rubra* is highly similar to *U. americana*, including a ciliate samara margin or pubescent samara body, which is totally different from sect. *Ulmus*. Besides, three species from sect. *Chaetoptelea* formed a polyphyletic group, in which *U. alata* and *U. thomasii* was clustered to sect. *Blepharocarpa* and sect. *Trichoptelea* respectively, and *U. elongata* was independent from them.

A satisfactory agreement for the backbone of *Ulmus* is found between the gene tree and species tree (Fig. 6). However, there are still several significant incongruences, which are mostly distributed in the shallow nodes with low or mid support (Fig. 4). Given that the backbone of *Ulmus* phylogeny is highly consistent between different dataset (i.e. cp genome, PCGs and noncoding regions) and methods (i.e. BI, ML) from cp genome, plastomic data is still sufficient for phylogenetic resolution of this genus. The prevalent conflicts have been focused on the five species from sect. *Ulmus* (Fig. 6), which may be caused by incomplete lineage sorting, network evolution and polyploidy. Within *Ulmus*, the extent of hybridization has been reported from population genetics [50, 51], and also inferred by inconsistent phylogeny between nuclear and cp genomes in the previous studies [9, 10]. The chromosomes number of *Ulmus* is relatively stable with 2n = 28 in most of species, which might rule out as the cause of ancient polyploidy [8]. In summary, phylogenomic analysis from nuclear datasets including genomes and transcriptomes are urgently needed for better understanding the species diversity and evolution for *Ulmus* in the future studies.

## Conclusions

Our current study provided comprehensive insights into the whole cp genome organization and content of *U. mianzhuensis*. Although the structure and gene content evolution of cp genomes are highly conserved within *Ulmus* species, we still found structural variations in IR/SC boundary at the section levels. Furthermore, important genetic characteristics including repetitive sequences, SSRs, and sequence divergence were explored to population genetics and DNA barcoding. We also reconstructed the phylogenomic relationship within *Ulmus* and provided an important foundation for further revision and adaptive evolution of *Ulmus.* In future, we will focus on conflicts between the gene tree and species tree with extensive samplings and nuclear datasets to verify the potential mechanism for the recent radiations in *Ulmus*.

## Materials and methods

### Ethical statement

For the collection of samples for this study, no special licenses were needed. The relevant Chinese laws were followed as this research was conducted.

### Taxon sampling, sequencing and assembly

Fresh leaves of *U. mianzhuensis* were collected from Central Forest Tree Nursery in Sichuan, China and stored in silica gel prior. The voucher specimen (Deng11468) was deposited in the Herbarium of Henan Agricultural University. For this species, 50 mg dried leaves were ground and high-quality genomic DNAs were extracted using a Plant Genomic DNA Extraction Kit (Tiangen, Beijing, China), and then were subsequently sent to Novogene (http://www.novogene.com, China) for short insert (350 bp) library construction and next-generation sequencing by Illumina Hiseq 4000 genome analyzer platform (Illumina, San Diego, CA) by Novogene, Beijing, China. Raw reads from the paired-end for quality were filtered with the NGSQC ToolKit by removing adapter sequences and low-quality reads with Q value B20 [52]. Then the clean data were assembled by NOVOPlasty 2.6.3 [53]and annotated by GeSeq [54], and the results were manually checked and verified in Geneious v.9.1 according to *Ulmus parvifolia* (MT165940) [55]. The visualization of the cp genome map was performed in OGDRAW [56].

### Comparisons analyses of cp genomes in *Ulmus* species

We obtained 31 cp genomes from NCBI representing all the five sections of *Ulmus* species (Table S1). The genome size GC content, gene content and number were firstly compared based on Geneious v.9.1 and manual correction [55]. Since the structure and organization of cp. genomes are highly conservative within sections, eight cp genomes were selected representing the five sections and different series to explore the genome variations among *Ulmus* species. We initially aligned all the complete cp genomes using MAFFT v7.0 and further checked by manual [57]. The cp genomes with annotation were compared by using the program mVISTA in shuffle-LAGAN mode with *U. parvifolia* as a reference [58]. We employed the IRscope script to generate and compared the variation of inverted-repeat (IR) and single-copy (SC) borders of the cp genomes from five sections. [59]. For simple sequence repeats (SSRs), we used MISA by setting the minimum number of repeats to 10, 6, 5 for mono-, di-, tri- repeats, and 3 for tetra-, penta-, and hexanucleotides repeats, respectively [60]. REPuter were used to identify and locate the repeat sequences with 30 bp minimum repeat size, 90% or greater sequence identity [61].

### Evaluation of variation hotspots

In addition, DnaSP v5.0 was employed to analyze nucleotide diversity (Pi) for variation hotspots among the *Ulmus* species [62]. A sliding window was conducted to compare Pi value based on a 600 bp window length with a 200 bp step size. We further divided the all protein-coding genes (PCGs) into 11 groups and also evaluated the Pi value at a functional group level for all protein-coding genes (PCGs) and quadripartite structure to detect nucleotide heterogeneity (Table S3).

### Phylogenomic analysis and adaptive evolution

A total of 32 species were used for our phylogenetic analysis including *U. mianzhuensis* and 30 relatives representing almost *Ulmus* species across the world, with *Zelkova schneideriana* (Ulmaceae) as outgroup (Table S1). For concatenated analysis, both cp genome and 79 protein-coding genes were compiled into a single file of the 32-taxon data set respectively and aligned with MAFFT v7.0 for phylogenetic inferences [57]. Maximum likelihood (ML) analyses were conducted using IQ-TREE [63]and the best substitution model and partitioning scheme were simultaneously implemented in ModelFinder [37] under the Bayesian information criterion (BIC) [64]. The Bayesian inference (BI) analysis was carried out using MrBayes v3.2 [65], and the Markov chain Monte Carlo analysis was executed for 100,000,000 generations, with four chains (one cold and three heated) sampled at every 1000 generations. The first 25% of the trees were discarded as burn-in, and the remaining trees were used to construct majority-rule consensus trees. The convergence of runs was estimated by Tracer v1.5 [66]. To further estimate a coalescent-based species tree, we first inferred individual gene trees using RAxML v8.2.11 under The GTRGAMMA model and with 1000 bootstrap replicates. The resulting gene trees were collapsed to infer a species tree with ASTRAL-III v5.6.3 [67] using local posterior probabilities to assess clade support. To detect the selective pressure of protein-coding genes in *Ulmus*, the sequences for each gene were aligned separately and the maximum likelihood phylogenetic tree from the protein-coding gene was used as a constraint tree. The synonymous (dS), non-synonymous (dN) nucleotide substitution rates and the dN/dS ratio (ω) were calculated using the codeml program of PAML v4.9 [68]. The pairwise dN and dS substitution rates between different taxa were calculated based on the custom selection model by setting CodonFreq prior as F3 × 4 model [69]. The dN/dS ratio was then calculated and compared at the 11 functional groups of all PCGs to detect evolutionary rate heterogeneity.

## Electronic supplementary material

Below is the link to the electronic supplementary material.

Supplementary Material 1

## Data Availability

The datasets generated and analyzed during the current study are available in the [NCBI database & NCBI SRA database] repository. The assembled cp. genome of *Ulmus mianzhuensis* is deposited in GenBank of NCBI under accession number OQ130025.1 (https://www.ncbi.nlm.nih.gov/nuccore/OQ130025.1). The raw sequencing data of *Ulmus mianzhuensis* cp. genome can be available in the NCBI SRA database under the accession number SRR22968959 (https://www.ncbi.nlm.nih.gov/sra/SRR22968959). The accession numbers for the remaining datasets used and analyzed in this study are listed in the Table S1.

## Abbreviations

cp genome: The complete chloroplast genome
LSC: Large single copy
SSC: Small single copy
IR(s): Inverted repeat(s)
