# Supplementary material for: The complete chloroplast genome of Ulmus mianzhuensis with insights into structural variations, adaptive evolution, and phylogenetic relationships of Ulmus (Ulmaceae)
Source: BMC Genomics. 2023 Jun 29;24:366. doi: 10.1186/s12864-023-09430-1 (PMC10308733; doi:10.1186/s12864-023-09430-1)

The complete chloroplast genome of *Ulmus mianzhuensis* with insights into structural variations, adaptive evolution, and phylogenetic relationships of *Ulmus* (Ulmaceae)

Nan Lin^1,2^, Rui Liu^1^, Yakun Wang^1^, Peng Guo^1,2^, Yihan Wang^1,2^, Yanpei Liu^1,2,*^, Fude Shang^1,2,*^

*^1^College of Life Science,* *Henan Agricultural University, Zhengzhou, China*

*^2^Henan Engineering Research Center for Osmanthus Germplasm Innovation and Resource Utilization, Henan Agricultural University, Zhengzhou, China*

^*^*Authors for correspondence*:

Yanpei Liu: liuyanpei2007@126.com

Fude Shang: sfd3933@163.com

Table S1 Chloroplast genomes characteristics of *Ulmus* species included in this study. *U. mianzhuensis* was the newly sequenced in this study, and other species were accessed from the National Center for Biotechnology Information database. Note: IR = inverted repeat; LSC = large single copy; SSC = small single copy.

| Species | Section | Genome size (bp) | LSC length  (bp) | IR length (bp) | SSC  length (bp) | GC content | GenBank accession  number |
| --- | --- | --- | --- | --- | --- | --- | --- |
| *U. alata* | sect. *Chaetoptelea* | 159353 | 87792 | 26406 | 18749 | 36.6 | MT165919 |
| *U. americana* | sect. *Blepharocarpus* | 159085 | 87600 | 26410 | 18665 | 35.6 | MT165920 |
| *U. bergmanniana* | sect. *Ulmus* | 159767 | 88193 | 26297 | 18980 | 35.5 | MT165921 |
| *U. canescens* | sect. *Ulmus* | 159187 | 87699 | 26376 | 18736 | 35.6 | MT165922 |
| *U. castaneifolia* | sect. *Ulmus* | 159700 | 88016 | 26361 | 18962 | 35.5 | MT165923 |
| *U. changii* | sect. *Ulmus* | 159376 | 87958 | 26330 | 18758 | 35.6 | MT165924 |
| *U. chenmoui* | sect. *Ulmus* | 159528 | 87938 | 26296 | 18998 | 35.5 | MT165925 |
| *U. crassifolia* | sect. *Microptelea* | 159338 | 87839 | 26413 | 18673 | 35.6 | MT165926 |
| *U. davidiana* | sect. *Ulmus* | 159645 | 88249 | 26297 | 18802 | 35.5 | MT165927 |
| *U. densa* | sect. *Ulmus* | 159322 | 87910 | 26324 | 18764 | 35.6 | MT165928 |
| *U. elongata* | sect. *Chaetoptelea* | 159165 | 87654 | 26410 | 18691 | 35.6 | MT165929 |
| *U. gaussenii* | sect. *Ulmus* | 159699 | 88015 | 26361 | 18962 | 35.5 | MT165930 |
| *U. glabra* | sect. *Ulmus* | 159305 | 87916 | 26348 | 18693 | 35.6 | MT165931 |
| *U. glaucescens* | sect. *Ulmus* | 159342 | 87973 | 26306 | 18757 | 35.6 | MT165932 |
| *U. laciniata* | sect. *Ulmus* | 159711 | 88118 | 26296 | 19001 | 35.5 | MT165933 |
| *U. laevis* | sect. *Blepharocarpus* | 159019 | 87529 | 26420 | 18650 | 35.6 | MT165934 |
| *U. lamellosa* | sect. *Ulmus* | 159722 | 88244 | 26297 | 18884 | 35.5 | MT165935 |
| *U. lanceifolia* | sect. *Ulmus* | 158742 | 87170 | 26404 | 18764 | 35.6 | MT165936 |
| *U. macrocarpa* | sect. *Ulmus* | 159684 | 88048 | 26299 | 19038 | 35.5 | MT165937 |
| *U. mianzhuensis* | sect. *Microptelea* | 159425 | 87584 | 26546 | 18749 | 35.6 | OQ130025 |
| *U. microcarpus* | sect. *Ulmus* | 159795 | 88408 | 26288 | 18811 | 35.5 | MT165938 |
| *U. minor* | sect. *Ulmus* | 159304 | 87915 | 26348 | 18693 | 35.6 | MT165939 |
| *U. parvifolia* | sect. *Microptelea* | 159233 | 87800 | 26317 | 18799 | 35.6 | MT165940 |
| *U. prunifolia* | sect. *Ulmus* | 159712 | 88028 | 26361 | 18962 | 35.5 | MT165941 |
| *U. pumila* | sect. *Ulmus* | 159685 | 88267 | 26288 | 18842 | 35.5 | MT165942 |
| *U. rubra* | sect. *Ulmus* | 159202 | 87717 | 26410 | 18665 | 35.6 | MT165943 |
| *U. serotina* | sect. *Trichoptelea* | 159270 | 87762 | 26413 | 18682 | 35.6 | MT165944 |
| *U. szechuanica* | sect. *Ulmus* | 159588 | 88035 | 26296 | 18961 | 35.5 | MT165945 |
| *U. thomasii* | sect. *Chaetoptelea* | 159457 | 87886 | 26413 | 18745 | 35.5 | MT165946 |
| *U. uyematsui* | sect. *Ulmus* | 159693 | 88116 | 26296 | 18985 | 35.5 | MT165947 |
| *U. wallichiana* | sect. *Ulmus* | 159422 | 87993 | 26368 | 18693 | 35.6 | MT165948 |

Table S2 The best nucleotide substitution and partitioned model for the chloroplast genome and the partitioned protein-coding genes (PCGs) datasets used in defined by ModelFinder.

| Datasets | best model from Bayesian information criterion | Best score |
| --- | --- | --- |
| chloroplast genome | TVM+F+R2: | -277419.835 |
| PCGs | K3Pu+F+I: (*accD+atpA+atpE+atpF+ndhH+petA+rpl14+rpl20+rpl32+rpoA+rpoB+rpoC1+rpoC2+rps15*+*rps16+rps18+rps19+rps2+rps3+rps4+rps8+ycf3*)  F81+F+I: (*atpB+clpP+psaC+rbcL+rpl16+rpl23+rpl2+rpl33+rpl36+rps11+rps12+rps14+rps7+ycf15*)  K3Pu+F: (*atpH+ndhJ+ndhK+petB+petD+petL+petN+psaA+psaB+psbA+psbB+psbC+psbD+psbE+psbF+psbH+psbI+psbJ*)  K3Pu+F:  (*atpI+ndhB+psaI+psbK+psbL+psbM+psbN+psbT+ycf2*)  K3Pu+F+I: (*ccsA+cemA+matK+ndhA+ndhC+ndhD+ndhE+ndhF+ndhG+ndhI+petG+psaJ+psbZ+ycf4*)  K3Pu+F+I:  (*rpl22+ycf1*) | -102939.545 |

Table S3 Functional groups identified for all protein-coding genes (PCGs) in the current study.

| Functional groups | Genes |
| --- | --- |
| Photosystem I (PSA) | *psaA*, *psaB*, *psaC*, *psaI*, *psaJ* |
| Photosystem II (PSB) | *psbA*, *psbB*, *psbC*, *psbD*, *psbE*, *psbF*, *psbH*, *psbI*, *psbJ*, *psbK*, *psbL*, *psbM*, *psbN*, *psbT*, *psbZ* |
| Cytochrome B6f complex (PET) | *petA*, *petB*, *petD*, *petG*, *petL*, *petN* |
| ATP synthase (ATP) | *atpA*, *atpB*, *atpE*, *atpF, atpH*, *atpI* |
| RNA polymerase (RPO) | *rpoA*, *rpoB*, *rpoC1*, *rpoC2* |
| Ribosomal proteins large subunit (RPL) | *rpl2*, *rpl14*, *rpl16*, *rpl20*, *rpl22*, *rpl23*, *rpl32*, *rpl33*, *rpl36* |
| Ribosomal proteins small subunit (RPS) | *rps2*, *rps3*, *rps4*, *rps7*, *rps8*, *rps11*, *rps12*, *rps14*, *rps15*, *rps16*, *rps18*, *rps19* |
| NADH dehydrogenase (NDH) | *ndhA*, *ndhB*, *ndhC*, *ndhD*, *ndhE*, *ndhF*, *ndhG*, *ndhH*, *ndhI*, *ndhJ*, *ndhK* |
| Conserved coding frame (CSF) | *ycf1*, *ycf2*, *ycf3*, *ycf4*, *ycf12* |
| **Other genes (OG)** | |
| Acetyl-CoA-carboxylase | *accD* |
| ATP-dependent protease | *clpP* |
| Cytochrome c biogenesis | *ccsA* |
| Membrane protein | *cemA* |
| Maturase | *matK* |
| Rubisco large subunit (Rubisco) | *rbcL* |

Table S4 The estimated substitution rates of all protein-coding genes (PCGs) in *Ulmus* species of this study.

| Genes | dN  (nonsynonymous) | dS  (synonymous) | dN/dS | Region | Group assigned |
| --- | --- | --- | --- | --- | --- |
| *accD* | 0.0235 | 0.0794 | 0.29667 | LSC | Other |
| *atpA* | 0.0026 | 0.0633 | 0.04117 | LSC | ATP |
| *atpB* | 0.0027 | 0.0171 | 0.15561 | LSC | ATP |
| *atpE* | 0 | 0.0444 | 0.0001 | LSC | ATP |
| *atpF* | 0.0166 | 0.0153 | 1.08398 | LSC | ATP |
| *atpH* | 0 | 0.0195 | 0.0001 | LSC | ATP |
| *atpI* | 0.0068 | 0.0219 | 0.31237 | LSC | ATP |
| *ccsA* | 0.0151 | 0.0818 | 0.18405 | SSC | Other |
| *cemA* | 0.0108 | 0.0519 | 0.20831 | LSC | Other |
| *clpP* | 0.0022 | 0.0306 | 0.07078 | LSC | Other |
| *matK* | 0.0187 | 0.0251 | 0.74297 | LSC | Other |
| *ndhA* | 0.0086 | 0.0493 | 0.17476 | SSC | NDH |
| *ndhB* | 0 | 0.0006 | 0.0001 | IR | NDH |
| *ndhC* | 0.0145 | 0.0568 | 0.25495 | LSC | NDH |
| *ndhD* | 0.0125 | 0.0866 | 0.14412 | SSC | NDH |
| *ndhE* | 0 | 0.0491 | 0.0001 | SSC | NDH |
| *ndhF* | 0.0313 | 0.1042 | 0.30038 | SSC | NDH |
| *ndhG* | 0.0115 | 0.0369 | 0.31115 | SSC | NDH |
| *ndhH* | 0.0057 | 0.0564 | 0.10144 | SSC | NDH |
| *ndhI* | 0.0116 | 0.1238 | 0.09358 | SSC | NDH |
| *ndhJ* | 0.0032 | 0.0371 | 0.08685 | LSC | NDH |
| *ndhK* | 0.0077 | 0.016 | 0.48057 | LSC | NDH |
| *petA* | 0.0096 | 0.0546 | 0.17613 | LSC | PET |
| *petB* | 0.002 | 0.0419 | 0.04883 | LSC | PET |
| *petD* | 0 | 0.0401 | 0.0001 | LSC | PET |
| *petG* | 0 | 0.1159 | 0.0001 | LSC | PET |
| *petL* | 0.0001 | 0.0002 | 0.4 | LSC | PET |
| *petN* | 0 | 0.0464 | 0.0001 | LSC | PET |
| *psaA* | 0.0022 | 0.0376 | 0.05968 | LSC | PSA |
| *psaB* | 0.0013 | 0.024 | 0.05345 | LSC | PSA |
| *psaC* | 0 | 0.0617 | 0.0001 | SSC | PSA |
| *psaI* | 0.0001 | 0.0001 | 0.4 | LSC | PSA |
| *psaJ* | 0 | 0.1206 | 0.0001 | LSC | PSA |
| *psbA* | 0.0012 | 0.0158 | 0.07639 | LSC | PSB |
| *psbB* | 0.0018 | 0.03 | 0.0603 | LSC | PSB |
| *psbC* | 0 | 0.0139 | 0.0001 | LSC | PSB |
| *psbD* | 0.0012 | 0.0202 | 0.01593 | LSC | PSB |
| *psbE* | 0 | 0.0142 | 0.0001 | LSC | PSB |
| *psbF* | 0 | 0.029 | 0.0001 | LSC | PSB |
| *psbH* | 0.0001 | 0.0001 | 0.74844 | LSC | PSB |
| *psbI* | 0 | 0.026 | 0.0001 | LSC | PSB |
| *psbJ* | 0 | 0.0838 | 0.0001 | LSC | PSB |
| *psbK* | 0.0001 | 0.0002 | 0.33747 | LSC | PSB |
| *psbL* | 0.0001 | 0.0002 | 0.3479 | LSC | PSB |
| *psbM* | 0 | 0.0537 | 0.0001 | LSC | PSB |
| *psbN* | 0.0095 | 0.0486 | 0.19642 | LSC | PSB |
| *psbT* | 0 | 0 | 0 | LSC | PSB |
| *psbZ* | 0.0071 | 0.0493 | 0.14482 | LSC | PSB |
| *rbcL* | 0.0179 | 0.0276 | 0.64909 | LSC | Other |
| *rpl14* | 0.0035 | 0.0166 | 0.20911 | LSC | RPL |
| *rpl16* | 0 | 0.1006 | 0.0001 | LSC | RPL |
| *rpl2* | 0.0066 | 0.0279 | 0.2378 | IR | RPL |
| *rpl20* | 0.0075 | 0.042 | 0.17958 | LSC | RPL |
| *rpl22* | 0.0001 | 0.0001 | 0.95308 | LSC | RPL |
| *rpl23* | 0 | 0.0276 | 0.0001 | IR | RPL |
| *rpl32* | 0 | 0.0275 | 0.0001 | SSC | RPL |
| *rpl33* | 0 | 0 | 0 | LSC | RPL |
| *rpl36* | 0 | 0.0937 | 0.0001 | LSC | RPL |
| *rpoA* | 0.0107 | 0.0499 | 0.21418 | LSC | RPO |
| *rpoB* | 0.0054 | 0.038 | 0.14168 | LSC | RPO |
| *rpoC1* | 0.0057 | 0.0422 | 0.13443 | LSC | RPO |
| *rpoC2* | 0.0151 | 0.035 | 0.43107 | LSC | RPO |
| *rps11* | 0.0034 | 0.0126 | 0.27172 | LSC | RPS |
| *rps12* | 0.0036 | 0.0459 | 0.07946 | LSC | RPS |
| *rps14* | 0.0047 | 0.0306 | 0.15354 | LSC | RPS |
| *rps15* | 0.0171 | 0.012 | 1.42056 | SSC | RPS |
| *rps16* | 0.019 | 0.1121 | 0.16995 | LSC | RPS |
| *rps18* | 0 | 0.012 | 0.0001 | LSC | RPS |
| *rps19* | 0.012 | 0.0243 | 0.49635 | IR | RPS |
| *rps2* | 0.0093 | 0.0495 | 0.18792 | LSC | RPS |
| *rps3* | 0.0108 | 0.059 | 0.18178 | LSC | RPS |
| *rps4* | 0.0022 | 0.0447 | 0.04928 | LSC | RPS |
| *rps7* | 0.0001 | 0.0001 | 0.0001 | IR | RPS |
| *rps8* | 0.0107 | 0.0116 | 0.92569 | LSC | RPS |
| *ycf1* | 0.0346 | 0.0769 | 0.44966 | IR | CSF |
| *ycf15* | 0 | 0 | 0 | IR | CSF |
| *ycf2* | 0.0039 | 0.0063 | 0.61996 | IR | CSF |
| *ycf3* | 0.0076 | 0.0352 | 0.21442 | LSC | CSF |
| *ycf4* | 0.0133 | 0.0594 | 0.22442 | LSC | CSF |

Figure S1 Genome alignment of eight representative *Ulmus* genomes from different section and series using mVISTA. Alignment was performed using *U. parvifolia* as a reference. Grey arrows above the alignment indicate the orientation of genes. Purple bars represent exons, blue ones represent introns, and pink ones represent non-coding sequences. A cut-off of 50% identity was used for the plots. The Y-scale axis represents the percent identity within 50-100%.


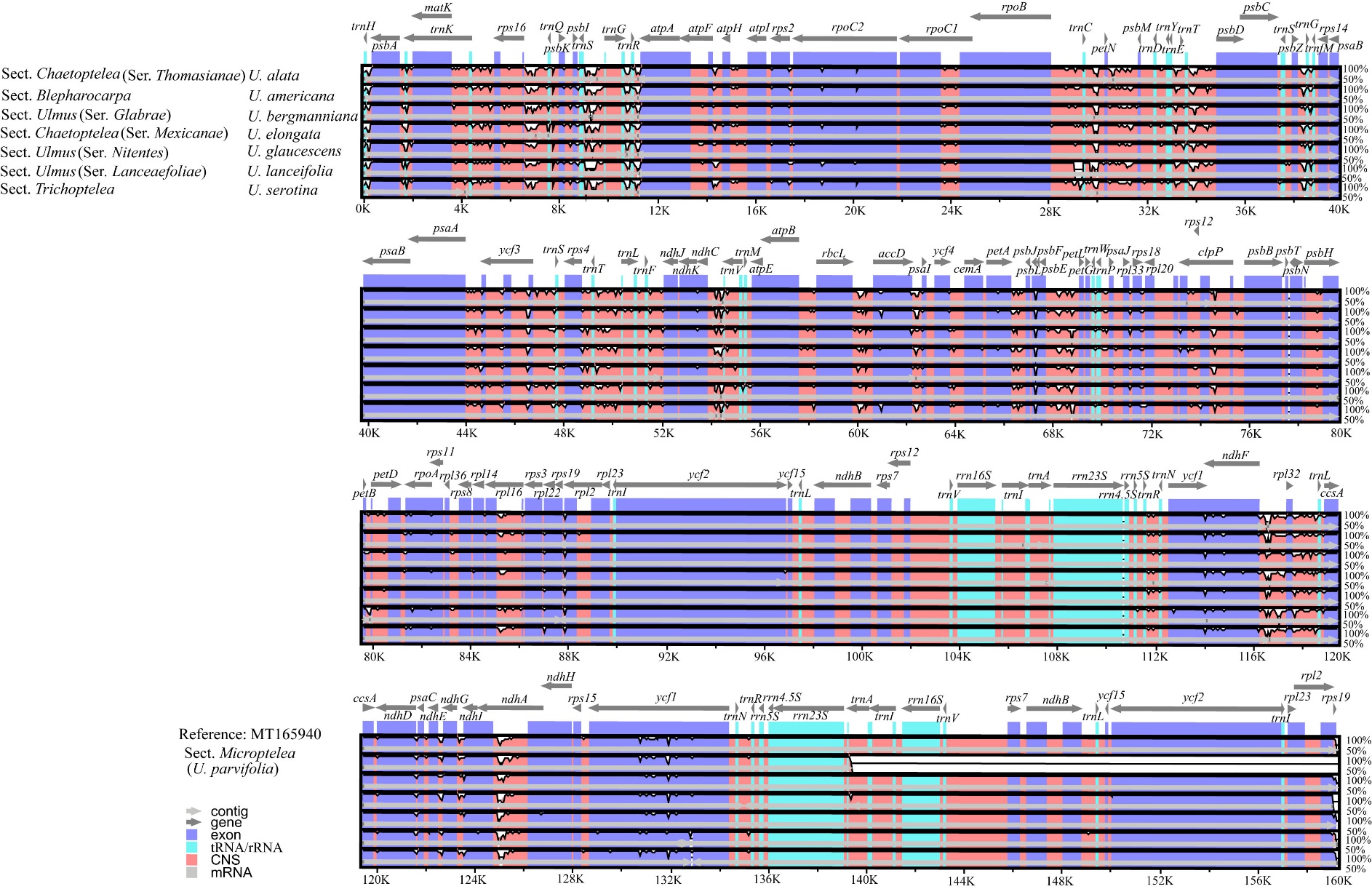

Supplement: Supplementary file 1 — Supplementary Material 1 [file 12864_2023_9430_MOESM1_ESM.docx]
